# DNA and mRNA vaccination against allergies

**DOI:** 10.1111/pai.12964

**Published:** 2018-09-20

**Authors:** Sandra Scheiblhofer, Josef Thalhamer, Richard Weiss

**Affiliations:** ^1^ Department of Biosciences University of Salzburg Salzburg Austria

**Keywords:** allergy, DNA vaccines, genetic vaccines, healthy responses, mRNA vaccines, natural protection, prevention, therapy

## Abstract

Allergen‐specific immunotherapy, which is performed by subcutaneous injection or sublingual application of allergen extracts, represents an effective treatment against type I allergic diseases. However, due to the long duration and adverse reactions, only a minority of patients decides to undergo this treatment. Alternatively, early prophylactic intervention in young children has been proposed to stop the increase in patient numbers. Plasmid DNA and mRNA vaccines encoding allergens have been shown to induce T helper 1 as well as T regulatory responses, which modulate or counteract allergic T helper 2–biased reactions. With regard to prophylactic immunization, additional safety measurements are required. In contrast to crude extracts, genetic vaccines provide the allergen at high purity. Moreover, by targeting the encoded allergen to subcellular compartments for degradation, release of native allergen can be avoided. Due to inherent safety features, mRNA vaccines could be the candidates of choice for preventive allergy immunizations. The subtle priming of T helper 1 immunity induced by this vaccine type closely resembles responses of non‐allergic individuals and—by boosting via natural allergen exposure—could suffice for long‐term protection from type I allergy.

## 1 INTRODUCTION

The processes following immunization with plasmid DNA or mRNA vaccines closely resemble those associated with viral infections; that is, within transfected cells, the encoded proteins are transcribed and translated and presented to the immune system. Hence, the assumption that these vaccines might be especially suitable to fight viral infections and tumors via cell‐mediated immunity was obvious. Indeed, early studies in animal models proved the efficacy of DNA vaccines in eliciting powerful cytotoxic T‐cell responses.^1^ However, it was also quite early recognized that the characteristic T helper 1 (TH1)–biased immune response alongside with production of IFN‐γ by CD4+ and CD8+ T cells elicited by this vaccine type could be utilized for modulating allergic T helper 2 (TH2) reactions, which are accompanied by secretion of the key cytokines IL‐4, IL‐5, and IL‐13, and allergen‐specific IgE.

The first studies investigating the anti‐allergic potential of DNA vaccines encoding clinically relevant allergens were performed with constructs encoding the major allergens from birch and house‐dust mite. In these experiments, it was proven that in animal models immunization with allergen‐encoding plasmid DNA itself induces a TH1‐biased response, while concomitantly avoiding production of IgE and is even able to counteract an established allergic TH2 reaction in a therapeutic setting.^2^

**Figure 1:**
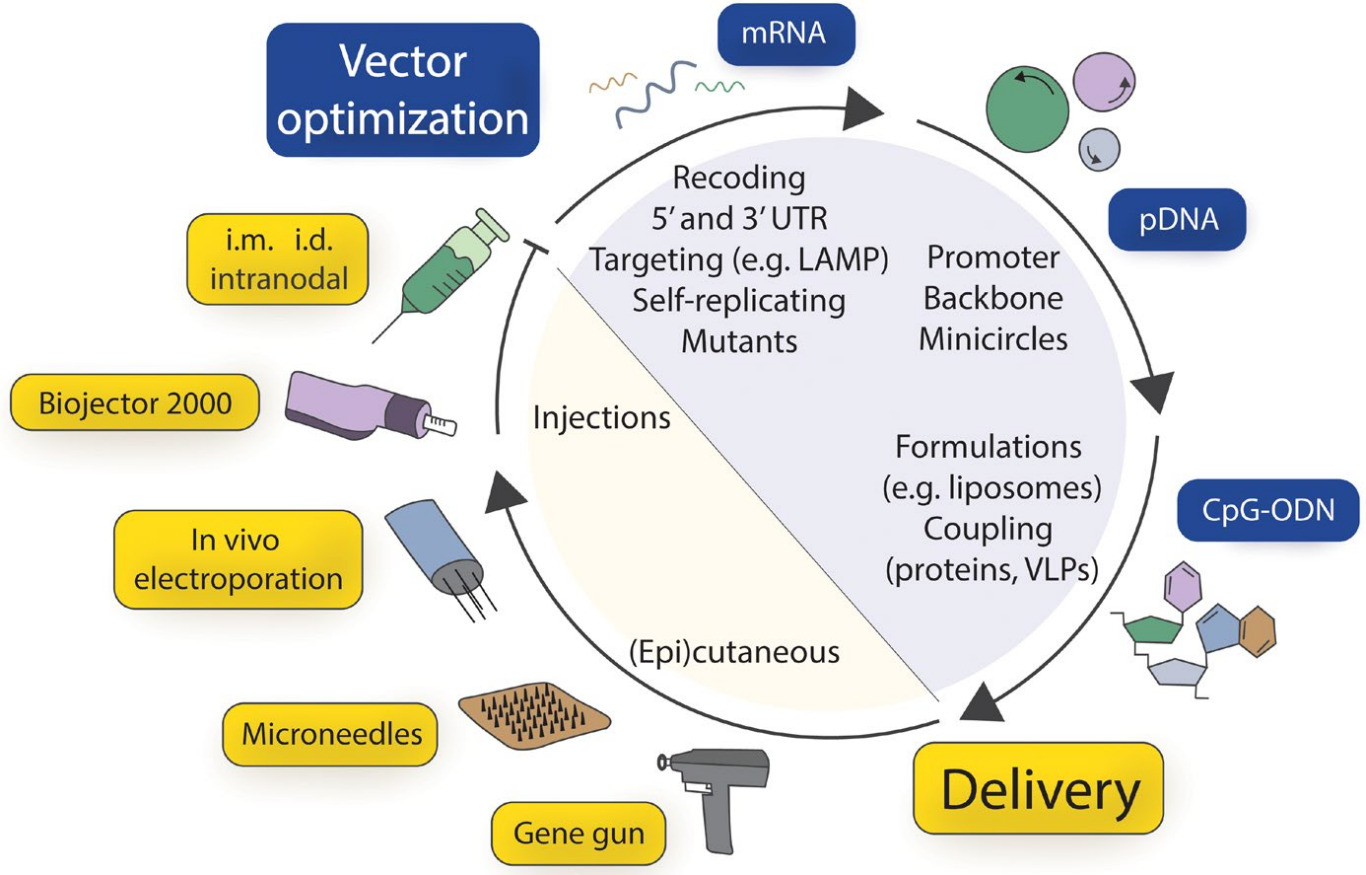
Optimization strategies for genetic vaccines. Modifications of the genetic sequence encoding the antigen of interest include (A) recoding in order to adapt the codon usage, adjust the GC content, and remove sequences that inhibit efficient translation (B) optimization of 5′ and 3′ UTRs to enhance mRNA stability and translation (C) usage of targeting sequences that shuttle the translated protein into specific cellular compartments (D) use of self‐replicating RNAs by incorporating alphavirus replicases and (E) mutations of the antigen itself to influence its immunogenicity and/or allergenicity. Such optimized sequences can be used directly as mRNA vaccines, or expressed from a plasmid DNA vector (pDNA). By choosing different promoters, expression strength and cell specificity can be adjusted. Using minicircle plasmids, unwanted bacterial sequences or antibiotic resistance genes needed for production can be removed from the plasmid backbone. Immunostimulatory CpG‐ODN can be covalently linked to an antigen of interest or incorporated in liposomal formulations or viruslike particles (VLP) to enhance their efficacy. Genetic vaccines can be injected intramuscularly (i.m.), intradermally (i.d., also using injection devices like the Biojector^™^ 2000) or even applied intranodal. Alternatively, (epi)cutaneous vaccination can be achieved with biolistic devices (gene gun) or using microneedles, with or without additional in vivo electroporation

After a veritable hype of DNA vaccines during the 1990s, when the efficacy of this vaccine type against a multitude of infectious diseases and tumors could be demonstrated in small animals, vaccination experiments in primates and clinical trials turned out to be rather sobering.^3^ Consequently, a variety of optimization strategies was developed over the following years (Figure 1). These include adaptation of the sequence to mammalian codon usage for enhanced expression, sophisticated delivery devices including gene gun, Biojector 2000, and microneedles, exploration of different application routes (intradermal, intramuscular, intranodal), the use of adjuvants such as CpG oligodeoxynucleotides (ODN), co‐immunization with vectors expressing immune‐enhancing or modulating chemokines and cytokines, and targeting of dendritic cells (DCs)^4^ or specific cellular compartments by the addition of certain targeting sequences.^2^, ^5^, ^6^, ^7^ Based on findings, which indicate that genetic vaccines might be useful in priming a broad immune response, also prime‐boost regimens combining genetic vaccines with protein or viral vectors have been explored.^9^ Furthermore, safety issues have been addressed by promoting the “revival” of mRNA vaccines^9^ and introducing self‐replicating (and thereby self‐limiting) DNA and mRNA vaccines based on alphaviral sequences.^10^

This review discusses the current status of DNA‐ and mRNA‐based vaccines against type I allergic reactions and provides an overview of recent and most promising concepts tested in the clinics.

## 2 EARLY APPROACHES

The first studies investigating the anti‐allergic effects of DNA immunization used simple conventional plasmids driving expression by a CMV promoter. Intramuscular immunization of rats with plasmid DNA encoding the house‐dust mite allergen Der p 5–induced specific immune responses, but prevented the formation of Der p 5–specific IgE. Moreover, pre‐vaccination with this construct prevented histamine release in bronchoalveolar lavage fluids and airway hyperresponsiveness upon challenge with aerosolized allergen. This effect was long‐lasting and transferable by CD8+ T cells from immunized to naïve animals.^11^ Subsequently, plasmid DNA constructs encoding a multitude of model allergens and clinically relevant molecules have been used for anti‐allergic immunization of rodents (reviewed in^2^).

Several of these early studies in mice have demonstrated a clear correlation between IFN‐γ secretion by T cells and production of IgG2a and protection from type I allergy by plasmid DNA vaccines.^11^, ^12^, ^13^, ^14^ Moreover, we could show that, in contrast to wild‐type animals, IFN‐γ, but not IL‐12p40–knockout mice failed to be protected from allergic sensitization by immunization with plasmids encoding grass pollen allergen.^15^ These findings underscore the central role of this cytokine in prevention of type I allergy.

## 3 INDUCTION OF BLOCKING IgG BY GENETIC VACCINATION

In rodents, antibody responses following immunization with genetic vaccines encoding allergens are dominated by the IgG2a subclass, which notably is able to activate complement. This clearly contrasts the situation in humans after SIT or SLIT, which mainly induce IgG4 antibodies lacking the capacity to fix complement. Allergen‐specific IgG2a antibodies can subsequently be boosted by exposure to the respective allergen as during sensitization or provocation via the airways. Furthermore, we could demonstrate that once induced, this humoral response type is long‐lasting and can be maintained even after repeated challenges with aerosolized allergen.^16^ With regard to IgG responses induced against allergens, the term “protective” conforms to the potential to block allergen‐IgE interactions. Activation of basophils from mice immunized with a DNA vaccine encoding Bet v 1 was shown to be increased following incubation with the allergen in the absence of antibody‐containing plasma, indicative of the presence of blocking IgG.^17^ To increase antibody responses in general and to generate blocking antibodies, it has been proposed that—besides efficient leader sequences in the plasmid constructs for secretion of allergen—techniques such as in vivo electroporation should be employed.^18^ In vivo electroporation following intramuscular DNA immunization against Der p 2 led to a substantial increase in IgG production, whereas the amount of IFN‐γ produced remained unaffected. However, it remains to be elucidated whether this improved humoral response contributes to the observed protective effect.^19^ In contrast to rodents, humans in general mount weak (antibody) responses after standard injection of genetic vaccines, most likely due to inefficient cellular uptake. Hence, methods to facilitate in vivo delivery of genetic vaccines have been employed, including gene gun vaccination, which induced protective antibody responses against hepatitis B in human non‐responders to conventional immunization.^20^ Currently, a clinical study investigating the immunogenicity of a DNA vaccine against hepatitis B combined with in vivo electroporation in subjects with chronic infection is underway (clinicaltrials.gov identifier: NCT02431312).

## 4 TAILOR‐MADE GENETIC VACCINES

Vaccination with plasmid DNA has been performed in many clinical studies without proven serious adverse events. However, general apprehensions about the safety of this vaccine type have been raised. These comprise (a) integration of the vaccine itself into the genome, which could lead to cancer development; (b) long‐term persistence of the administered plasmid DNA, potentially triggering production of anti‐DNA antibodies and thus leading to autoimmunity; and (c) long‐term expression of the encoded antigen, thereby causing systemic immunological effects such as a generalized inflammatory milieu. As a consequence, the FDA in the United States and the Paul Ehrlich Institute in Germany have categorized vaccination with plasmid DNA as gene therapy.^21^ Also allergy‐specific concerns have been raised; primarily that uncontrolled expression of natural allergens could potentially cause adverse events by cross‐linking of preexisting IgE. To avoid the before‐mentioned problems, safety‐optimized genetic vaccines have been established (Figure 1).

Detailed knowledge about structural features of major allergens including their immunodominant B‐ and T‐cell epitopes enables the generation of DNA vaccines encoding so‐called hypoallergenic molecules, which no longer bind specific antibodies, but retain their T‐cell epitopes. In this case, side effects associated with binding of IgE to native allergen can be avoided during therapy, while enabling efficient recruitment of allergen‐specific TH1 cells. Approaches to meet these requirements include fragmentation of allergen,^22^ introduction of point mutations,^22^ and the use of epitope vaccines encoding known CD4+ and/or CD8+ T‐cell epitopes.^23^, ^24^ Whereas DNA vaccines encoding two hypoallergenic fragments or a hypoallergenic mutant of the birch pollen allergen Bet v 1 both protected mice from sensitization with the allergen, only the fragment vaccine was able to reverse an established Th2‐type response. Notably, the fragment vaccine did not induce Bet v 1–specific antibodies indicative of differences in the three‐dimensional structure of the encoded fragments and the wild‐type allergen.^22^ With regard to DNA vaccines encoding epitopes, results are contradictory: While in some studies increased levels of IgG2a and IFN‐γ together with suppression of IgE upon sensitization was noted,^24^ in other cases these vaccines could not convincingly demonstrate their anti‐allergic potential.^23^

Alternatively, genetic vaccines, which also avoid release of large amounts of native allergen, can be created by the addition of certain targeting sequences to the encoded allergen. One possibility is targeting the allergen for proteasomal degradation by forced ubiquitination.^25^ The generated peptides are then either presented on MHC‐I, thereby facilitating the induction of CD8+ T cells (which have been shown to counteract allergic responses as producers of IFN‐γ) or directly get loaded onto MHC‐II molecules by a process termed autophagy. Alternatively, the encoded allergen can be targeted to the endolysosomal compartment by the addition of LIMP (lysosome membrane protein) or LAMP (lysosomal‐associated membrane protein) sequences. The resulting peptides are subsequently loaded onto MHC‐II molecules. Both approaches have in common that no native allergen is secreted, which could cross‐link preexisting IgE.

With regard to preventive immunization of healthy, young individuals against allergic sensitization, safety measurements have to be even stricter. However, using mRNA vaccines, the required safety profile could potentially be warranted. This vaccine type utilizes minimal vectors, which basically consist of the gene of interest, a poly‐adenosine tail of more than 30 residues at the 3′ end and a 7‐methylguanosine cap structure at the 5′ end. Additionally, untranslated regions (UTR) at the 3′ and/or 5′ end have been employed to enhance mRNA stability and thus increase protein translation. mRNA vaccines cannot integrate into the host genome and are readily cleared from the organism by RNAses. Furthermore, there has been no single report on formation of antibodies against mRNA molecules linked to autoimmune diseases. In 1990, naked mRNA was injected into muscle of mice and found to result in expression of different reporter molecules.^26^ Administration of a liposome‐formulated mRNA vaccine encoding the influenza virus nucleoprotein into mice induced virus‐specific cytotoxic T‐cell responses.^27^ Unexpectedly, also unprotected mRNA was found to elicit powerful humoral and cellular immune responses.^28^ Owing to this and their unsurpassed inherent safety features, this type of genetic vaccine has been repeatedly employed in clinical trials mainly in the cancer field. So far, immunotherapy with mRNA has been performed in patients suffering from melanoma, non‐small‐cell lung cancer, renal cell carcinoma, and prostate cancer.^21^, ^29^, ^30^ Recently, a protective mRNA vaccine against rabies was successfully tested in healthy subjects.^31^ Based on a study demonstrating that immunization with mRNA preferentially induces a TH2‐biased T‐helper cell polarization, which could only be redirected toward an anti‐allergic TH1 response by the addition of adjuvants,^32^ it was initially supposed that mRNA would not be suitable for counteracting allergic reactions. However, in the meantime mRNA vaccines have proven to be equally potent inducers of TH1 responses without adjuvantation compared to their DNA counterparts.^33^, ^34^ Notably, the immunogenicity of allergen‐encoding mRNA appeared to be reduced compared to DNA, at least for the grass pollen allergen Phl p 5. However, obviously even weak TH1 priming was sufficient for expansion of protective TH1 cells upon exposure to the specific allergen as mice were protected from formation of specific IgE.^34^ This process resembles the recall responses observed in vaccinated individuals after contact with infectious agents. In individuals vaccinated against allergen(s), subtle TH1 priming and recruitment of TH1 cells could provide long‐term protection as boosting of this immunity would naturally occur via exposure to allergen such as during the pollen season. Indeed, we could demonstrate sustained alleviation of airway hyperresponsiveness and reduction in lung eosinophilia under repeated exposure to aerosolized allergen.^16^

Besides targeting subcellular compartments, efforts have been made to generate DNA vaccines that express proteins targeted to DCs with the aim to enhance specific immune responses (reviewed in^35^). With respect to allergy DNA vaccines, recent studies found that targeting an allergen to DCs by encoding DEC‐205 fusion proteins alleviates allergic asthma in mice.^36^, ^37^ Interestingly, although non‐targeted allergen also protected from sensitization^37^, the latter was associated with a TH1 phenotype, while DC targeted vaccination induced reduced levels of IFN‐γ production^36^, ^37^ but was associated with elevated numbers of CD4 + CD25 + FoxP3 + T cells^37^. Surprisingly, both TH‐1 as well as regulatory T‐cell induction were equally efficient in suppressing allergic lung inflammation, highlighting that multiple mechanisms can confer allergy protective immune responses after DNA immunization.

## 5 MIMICKING THE HEALTHY STATUS

Since the discovery of IL‐10‐producing T regulatory 1 (Tr1) cells, also in the field of allergen‐specific immunotherapy, the main focus was set on this immune cell type, whereas immune modulation toward TH‐1 responses has been seen more and more critical. With regard to the TH‐1‐promoting potential of genetic vaccines, concerns have been raised that this response type could lead to exaggerated inflammation, in particular of the airways, and even autoimmunity. However, we and others found no pathological inflammation following genetic immunization, pointing to a self‐limiting capacity of TH‐1 cells via autologous IL‐10 secretion.^38^ Even after repeated monthly exposure to aerosolized allergen, an initially set protective TH‐1 bias did not end up in pathological TH‐1‐mediated inflammation.^16^ Alongside with the above‐mentioned importance of modulating the allergen‐driven TH‐2 response toward TH‐1, we and others also observed induction of regulatory T cells following immunization with plasmid DNA.^17^, ^39^ Likewise, protection did not necessarily correlate with total levels of IFN‐γ^17^, suggesting that besides IFN‐γ production other mechanisms might play a role in protection from allergic responses after plasmid‐based immunization.

According to the hygiene hypothesis, exposure to a traditional farming environment, that is, contact with farm animals and hay as well as consumption of non‐pasteurized milk early in life, protects from development of allergies.^40^ Specifically, children exposed to such a diversity of microbial compounds have lower frequencies of asthma, hay fever, and atopic sensitization.^41^ Furthermore, the protective “farming effect” also leads to a reduced risk for atopic sensitization in adulthood.^42^ Obviously, certain microbial products provide a strong activating stimulus for the innate immune system leading to an immune status counteracting allergic reactions.^41^ To investigate the mechanisms underlying the “farming effect” mice bred in a cattle barn or a conventional animal facility were sensitized with allergens via the skin and the resulting immune responses were analyzed.^43^ Compared to the conventionally raised animals, the farm mice developed an elevated number of activated CD4+ T cells early in life. Their cytokine profile was skewed toward IL‐17 and IL‐22 accompanied by increased IL‐10 secretion. It was concluded that the farming environment provides a strong, allergy protective IL‐22 stimulus, which might also provide protection from contact skin allergy.

To elaborate how the immune system of healthy, non‐allergic individuals deals with allergens, we studied a large cohort of donors and—in line with a recently postulated concept^44^—found that non‐allergic subjects do not display immunological ignorance, but react by mounting “healthy” immune reactions.^45^ These have been shown to be associated with regulation of epithelial barrier function^46^ as well as T‐cell responses.^47^, ^48^ When we expanded allergen‐specific T memory cells from peripheral blood mononuclear cells, we detected a broad spectrum of TH response types, which counteract allergic reactions. In a small percentage of subjects, we even found allergen‐specific Th17/TH22 responses presumably contributing to regulation of TH2 responses. Moreover, we could demonstrate that the prevalence of certain response types depends on the living environment by comparing responses of healthy individuals with a traditional farming background to those of townspeople. Notably, allergen‐specific T cells from townspeople displayed an even more pronounced tendency toward pro‐inflammatory responses. Based on our data, variable and complex immune deviation mainly toward a pro‐inflammatory phenotype can be assumed to confer natural protection from allergy.^44^ Genetic vaccines—and especially mRNA vaccines—might have the potential to deliver exactly this naturally occurring immunity.

## 6 PROPHYLAXIS VS THERAPY

In therapeutic animal models of allergy, genetic vaccines have proven their efficacy in counteracting TH2 responses by suppression of IgG1, IgE and allergic lung inflammation and by induction of IFN‐γ, and IgG2a. However, to modulate or even convert an established immune response is way more difficult than directing the developing immune system toward the desired protective pathways. Hence, repeated administration of high doses of the respective vaccine is required for only moderate downregulation of allergic symptoms.^7^ On the contrary, in mice suboptimal vaccine doses, which even fail to induce measurable immune responses, have been shown to lead to anti‐allergic reactions following provocation with allergen.^19^, ^34^ Also in humans barely detectable immune responses, as elicited by vaccination with mRNA vaccines, might be sufficient for maintenance of lifelong protection from allergy without booster immunizations. After the initial immunological bias is set, this is guaranteed due to natural exposure to the respective allergen(s).^6^ Clearly, testing prophylactic allergy vaccinations in the clinics would require careful selection of young children with a highly predictable risk to develop type I allergy. Besides adducing family anamnesis, diagnosis of certain food allergies during the first years of life,^49^ and of allergic reactions to so‐called primary sensitizers,^50^ identification of susceptibility genes for allergy and asthma^51^ provides a basis for delimitation of candidate individuals.

Whereas genetic vaccines for allergy prevention most likely would act by inducing pro‐inflammatory immune reactions, preventive treatment of children by oral or sublingual administration of allergen(s) was already performed with the aim to induce regulatory responses. Sublingual administration of a mixture of allergen extracts from house‐dust mite, cat, and timothy grass to high‐risk children at an age of 12‐30 months failed to induce regulatory T cells against the allergens delivered via the mucosa.^52^ The authors speculated that instead of promoting tolerance, the intervention did not trigger immunologic processes at all or only worked as a weak booster. As infants are not capable of holding the allergen drops under the tongue for up to 3 minutes, the allergen concentration at the mucosal surface might have been too low and the exposure time too short for tolerogenic mucosal DCs to acquire enough allergen. Oral administration of house‐dust mite extract in high‐risk children below 1 year of age lacking skin test reactivity to common allergens resulted in reduced sensitization to any common allergen, but not to house‐dust mite.^53^ In contrast, 2‐ to 5‐year‐old children sensitized against house‐dust mite and/or grass pollen treated by sublingual immunotherapy with the respective extract showed an upregulation of specific IgG and IL‐10‐associated function of regulatory T cells in vitro, whereas specific IgE and skin prick test reactivity remained comparable to the placebo group.^54^ These data indicate that induction of allergen‐specific regulatory T‐cell responses sufficient to prevent allergic sensitization is a difficult task and that additional “pro‐inflammatory” approaches including genetic vaccination and the use of immunomodulators such as CpG should also be taken into consideration. Notably, for prevention of allergies timing of intervention seems to be crucial: High‐risk infants between 4 and 11 months of age consuming at least 6 g of peanut protein per week until 60 months displayed a significantly reduced rate of peanut allergy compared to those avoiding peanuts.^55^ It has been suggested that using defined recombinant allergens or derivatives thereof combined with optimized dosage and timing for oral tolerance induction in children could provide a powerful measurement in allergy prevention.^56^

## 7 CURRENT CLINICAL DEVELOPMENTS

The company Immunomic Therapeutics recently developed a DNA vaccine by incorporating the sequence of lysosomal‐associated membrane protein 1 (LAMP‐1) into plasmids encoding the major allergens Cry j 1 or Cry j 2 from Japanese red cedar (JRC) pollen. Immunization of BALB/c mice with these constructs resulted in TH1‐biased immune responses as indicated by high levels of IFN‐γ and anti‐Cry j 1 or anti‐Cry j 2 IgG2a antibodies and low levels of IgE. Adoptive transfer of T cells from immunized into naïve mice followed by Cry j 1/Cry j 2 protein boosts revealed that CD4+ T cells are the immunological effectors of DNA immunization in this allergy model.^57^ Based on these data, a series of clinical trials was initiated (Table 1). Individuals sensitized to Japanese red cedar and/or mountain cedar were immunized four times at 14‐day intervals with 2 or 4 mg, respectively, of the plasmid encoding Cry j 2 linked to LAMP‐1 (JRC‐LAMP‐Vax). Immunizations were well tolerated by all participants. Interestingly, 130 days after the 4th immunization 10 of 12 patients sensitized to Japanese red cedar and six of 11 patients sensitized to mountain cedar displayed a conversion of skin test from positive to negative.^58^ However, as skin prick tests at the time‐point of enrollment and at the study end‐point were performed using allergen extracts, it is difficult to assess whether treatment induced any Cry j 2‐specific immunological effects. Notably, three of three subjects tested positive for Cry j 2 at screening were found to be skin test negative for Cry j 2 on day 132.

**Table 1:** Clinical trials using the LAMP‐Vax platform against type I allergies

Vaccine	Allergen (source)	Regimen	Method/Route	Dose	Phase	Identifier No	Reference
CryJ2‐LAMP DNA	Cry j 2 (Japanese red cedar)	4 × 14‐d intervals	i.m. injection	2 mg or 4 mg	Ia	NCT01707069	58
CryJ2‐LAMP DNA	Cry j 2 (Japanese red cedar)	1× (continuing Ia)	i.m. injection	2 mg	Ib	NCT01966224	58
CryJ2‐LAMP DNA	Cry j 2 (Japanese red cedar)	4 × 14‐d intervals	i.d. Biojector 2000	1.08 mg or 2.16 mg	Ic	NCT02146781	
ASP4070^a^	Cry j 2 (Japanese red cedar)	1× or 4×	i.d. or i.m.	“high or low”	I	NCT02469688	
ASP4070^a^	Cry j 2 (Japanese red cedar)	14‐d intervals	i.d.	“high or low”	II	NCT03101267	
ASP0892^b^	Ara h 1, 2, 3 (peanut)	4 × 14‐d intervals	i.d. or i.m.	“high or low”	I	NCT02851277	

i.m., intramuscular; i.d, intradermal.

a Also known as JRC (Japanese red cedar)‐LAMP‐Vax.

b Also known as ARA‐LAMP‐Vax.

Approximately 300 days after the first vaccination, some of the study participants received one booster dose of 2 mg to assess long‐term safety and recall immune responses. Interestingly, a substantial number of prick test conversions from positive to negative were also noted for unrelated allergens pointing to a possible bystander suppressive effect provided by T helper cells.^59^ Specific IgE titers remained unchanged upon immunization with the Cry j 2‐LAMP vaccine and unlike in the preceding mouse study, only a marginal increase in specific IgG was noted.^58^

As an alternative to intramuscular injections, in a follow‐up study this vaccine was also delivered intradermally using the Biojector^™^ 2000 device (clinicaltrials.gov identifier: NCT02146781). In an ongoing phase I trial (clinicaltrials.gov identifier: NCT02851277), a multivalent peanut‐LAMP‐1 DNA vaccine including Ara h 1, Ara h 2, and Ara h 3 is evaluated in peanut allergic patients by intradermal (undisclosed “low or high” dose) or intramuscular injections (undisclosed “high” dose; Table 1).

The LAMP‐Vax platform utilizes an up‐to‐date targeting approach, which should avoid therapy‐induced side effects caused by high amounts of free allergen. Alternatively, the synthesized allergen‐LAMP fusion protein is directly shuttled into the lysosomal compartment, circumventing exposure of the patient to native allergen. Instead of aiming to induce tolerance, this therapy is designed to reverse the allergenic IgE/TH2 response toward an IgG/TH1 response (https://www.immunomix.com/technology/allergy/).

Another approach, which utilizes non‐methylated CG‐rich DNA sequences (CpG‐motifs) either as adjuvant or even as unspecific treatment, has already proven its suitability in allergen‐specific immunotherapy. These sequences are common in bacteria, but are mostly methylated in the genome of vertebrates. CpGs bind to Toll‐like receptor 9 (TLR9) located in the endosomes of plasmacytoid DCs and monocyte‐derived DCs (mDCs) in mice, but only of plasmacytoid DCs in humans as well as of B cells.^60^ TLR9 signaling has been shown to promote TH‐1 immune responses in mice^61^ and humans^62^; hence, CpGs appear attractive for use as adjuvants in specific allergen immunotherapy. Due to differences in their structure, type A and B CpGs exert distinct immunological activities: A‐type CpGs contain their motifs in a palindromic form and their backbone consists of phosphodiester bonds, whereas the backbone of B‐type CpGs is chemically stabilized by phosphorothioate bonds and they do not form palindromes. Whereas A‐type CpGs have been identified as producers of type I interferons, B‐type CpGs essentially induce IL‐12 secretion.

Most early studies in mice have shown that CpGs can protect from allergic asthma by increasing levels of IFN‐γ and IL‐12 resulting in suppression of IL‐5 and concomitant lung eosinophilia.^63^ More recent data revealed that this reduction in lung inflammation is also accompanied by an upregulation of IL‐10 and CD4 + Foxp3 + T cells in the lung, indicating that CpG‐induced suppression of allergic inflammation may also contain a regulatory component.^64^ Already in 2006, ragweed allergen Amb a 1 covalently coupled to type B CpG‐ODNs was employed for treatment of ragweed allergic patients, who received six injections at weekly intervals (Table 2). Treatment was well tolerated and no severe side effects occurred.^65^ This could be explained by a reduced IgE reactivity of the coupled Amb a 1 as shown by histamine release from human basophils.^61^ Whereas vascular permeability determined via measurement of serum albumin concentrations in nasal lavages remained unaffected, symptom and quality‐of‐life scores were improved during the first and also the second pollen season after therapy. Interestingly, the treated patients did not mount an increase in IgE titers as usually observed shortly after the pollen season. However, a follow‐up phase II clinical study had to be terminated due to very mild symptoms of the placebo control group in the relevant pollen season, making measurement of a treatment effect impossible (clinicaltrials.gov identifier: NCT00387738). In humans, the type B CpG‐ODN used in these studies are potent stimulators of B cells and promote maturation and activation of plasmacytoid DCs, but induce only small amounts of IFN‐α/β. Furthermore, in contrast to mice, where type B CpGs induce IL‐12 production by mDCs, human mDCs do not express TLR9. Hence, inefficient stimulation of human DCs by B‐type CpGs might provide an explanation for the observed low clinical efficacy of the Amb a 1‐CpG conjugates.

**Table 2:** Clinical trials with CpG‐ODN against type I allergies

Vaccine	Allergen (source)	Regimen	Method/Route	Dose	Phase	Identifier No	Reference
AIC (TOLAMBA^™^)	Amb a 1 (ragweed)	6 × 1‐wk intervals	s.c. injection	escalating (3‐30 μg)	II	NCT00537355	65
AIC (TOLAMBA^™^)	Amb a 1 (ragweed)	6 × 1‐wk intervals	s.c. injection	escalating (“dose intense/low dose”)	II	NCT00387738	
QβG10 + HDM	Extract (HDM)	6 × 1‐wk intervals	s.c. injection	300 μg	I/IIa	NCT00652223	66
QβG10	No allergen	6 × 1‐wk intervals	s.c. injection	500 or 1000 μg	IIb	NCT00800332	68
QβG10	No allergen	7 × 1‐wk intervals	s.c. injection	900 μg	IIa	NCT00890734	74
QβG10	No allergen	7 × 1‐2‐wk intervals	s.c. injection	300, 1000, or 2000 μg	IIb	NCT01673672	75

AIC, Amb a 1 conjugated to CpG‐B DNA; HDM, house‐dust mite; s.c., subcutaneous; ODN, oligodeoxynucleotides; QβG10, CpG‐A ODN encapsulated in Qβ viruslike particle.

With the aim to stabilize and protect A‐type CpG‐ODN from digestion by DNAse I, they were encapsulated into viruslike particles (VLP) derived from bacteriophage Qβ. The CpG containing VLPs, termed QβG10, were then mixed with house‐dust mite extract, and this formulation was tested in phase I/IIa clinical trial with house‐dust mite allergic patients. Therapy was started with extract only administered subcutaneously at a standard dose‐escalating cluster regimen, that is, increasing doses at 30‐minutes intervals in two sessions with 1 week in between. Subsequently, treatment with the extract mixed with QβG10 was performed six times at weekly intervals.^66^ Conjunctival provocation tests revealed almost complete tolerance. The treatment significantly reduced rhinitis and asthma symptoms and this improvement lasted at least until 38 weeks after finishing the therapy. Injections of QβG10 and house‐dust mite extract induced an increase in allergen‐specific IgG and in IgE, the latter being only transient.

A follow‐up study was performed in which additional groups of patients were treated with house‐dust mite extract alone or QβG10 alone.^67^ Surprisingly, clinical effects observed in the QβG10 group were comparable to that of the QβG10 plus extract group. Encouraged by these results, a phase IIb trial was accomplished in a large cohort of patients receiving QβG10 at a high or a low dose, respectively, without allergen.^68^ Significant improvement of combined symptom and medication scores were monitored in the high‐dose group compared to placebo. In conjunctival provocation tests a 10‐fold increase in allergen tolerance was observed. How this unspecific allergy treatment is able to ameliorate allergic symptoms is not entirely understood. With a size of 30 nm, the VLPs get readily transported to draining lymph nodes,^69^ where they are phagocytosed and their CpG content can directly be delivered to the endolysosomes of plasmacytoid DCs.^70^ Upon binding of CpGs to TLR9, TH‐1‐promoting and simultaneously TH‐2‐suppressing cytokines are produced.^71^ In mice, it has been shown that CpG can ameliorate the asthmatic airway response by upregulating the activity of IDO (indoleamine 2,3 dioxygenase) in DCs leading to suppression of T cells.^72^ A direct effect of CpG‐ODN alone also perfectly fits to the hygiene hypothesis as stimulation of innate immunity by bacterial products is known to counteract allergic reactions. This is in line with our findings that healthy farmers show a tendency to mount allergen‐specific regulatory responses, whereas townspeople preferentially develop inflammatory reactions.^45^ However, CpGs could also directly act on mast cells as these also express TLR9.^73^

Undergoing controlled glucocorticosteroid withdrawal, patients with mild‐to‐moderate persistent allergic asthma were treated with the same accumulating dose of QβG10 in another clinical study. Significant improvement of all patient‐reported parameters and a significantly higher forced expiratory volume compared to placebo was recorded.^74^ However, when patients were treated in a further study with this compound, no significant differences between verum and placebo could be detected.^75^ In this case, QβG10 turned out to be ineffective in patients with uncontrolled moderate‐to‐severe allergic asthma as add‐on therapy to inhaled corticosteroids plus long‐acting beta‐agonists.

## 8 CONCLUSIONS

Unspecific treatment as with CpG DNA lacking any allergen preparation or extract would provide the opportunity to treat different allergies with a single compound. Besides the failure to demonstrate therapeutic efficacy of this approach in certain groups of patients, it has to be kept in mind that such an allergen‐free treatment might only work for allergies against ubiquitous and perennial allergens like those derived from house‐dust mite. In this case, permanent contact with allergen(s) might contribute to the therapeutic effect.

Theoretically, targeting of the encoded allergen to the lysosomal compartment as provided by the LAMP‐Vax platform should prevent release of native allergen into the circulation, avoiding therapy‐induced side effects. By addressing the endosomal/lysosomal compartment, enhanced MHC‐II presentation and the induction of powerful CD4+ T‐cell responses are induced. The low number of immunizations leading to reversion of skin tests appears promising. It has to be clarified in ongoing and future clinical trials whether the barely detectable increase in allergen‐specific IgG titers following this intervention can be boosted by natural exposure of vaccinated subjects to the respective (pollen) allergen.

Whereas the recently or currently evaluated DNA‐based vaccines in the clinics are designed for therapeutic interventions, potentially the greatest strength of this vaccine type would be proven by prophylactic vaccination of healthy young individuals. However, the safety and efficacy of genetic vaccines encoding allergens will have to be demonstrated in healthy as well as sensitized adults beforehand. Clinical trials investigating these issues have been performed or are currently underway. Highly probable, prophylactic as well as therapeutic genetic vaccines of choice will have to be rendered hypoallergenic; that is, no native allergen is secreted, thereby avoiding de novo IgE production (preventive immunization) or cross‐linking of pre‐formed IgE (therapeutic immunization). Moreover, candidate vaccines will most likely be designed to target certain types of immune cells. This can be either CD4+ T cells, which are most potent in preventing or modulating allergic TH2 responses or DCs, known for their ability to modulate and regulate immune responses. The former can be achieved by transporting the expressed allergen into subcellular compartments via addition of targeting sequences, the latter by formulations addressing specific receptors on DCs. Finally, due to their inherent safety features, mRNA vaccines could represent the ideal candidates to fulfill the requirements for prophylactic immunization against type I allergies.
